# Genetic Diversity of the Endangered Neotropical Cichlid Fish (*Gymnogeophagus setequedas*) in Brazil

**DOI:** 10.3389/fgene.2018.00013

**Published:** 2018-02-02

**Authors:** Lenice Souza-Shibatta, Thais Kotelok-Diniz, Dhiego G. Ferreira, Oscar A. Shibatta, Silvia H. Sofia, Lucileine de Assumpção, Suelen F. R. Pini, Sergio Makrakis, Maristela C. Makrakis

**Affiliations:** ^1^Laboratório de Genética e Ecologia Animal, Departamento de Biologia Geral, Universidade Estadual de Londrina, Londrina, Brazil; ^2^Laboratório de Genética e Conservação, Universidade Estadual do Norte do Paraná, Cornélio Procópio, Brazil; ^3^Museu de Zoologia, Departamento de Biologia Animal e Vegetal, Universidade Estadual de Londrina, Londrina, Brazil; ^4^Grupo de Pesquisa em Tecnologia em Ecohidráulica e Conservação de Recursos Pesqueiros e Hídricos – GETECH, Universidade Estadual do Oeste do Paraná, Toledo, Brazil

**Keywords:** conservation, freshwater, Lower Iguaçu River, *Gymnogeophagus*, endangered species

## Abstract

*Gymnogeophagus setequedas* is a rare and rheophilic species of tribe Geophagini, considered endangered in Brazilian red lists. Its previously known geographical distribution range was the Paraná River basin, in Paraguay, and a tributary of the Itaipu Reservoir in Brazil. Since its description no specimens have been collected in the original known distribution area. However, recent records of *G. setequedas* in the lower Iguaçu River, in a region considered highly endemic for the ichthyofauna, extended the known geographical distribution and may represent one of the last remnants of the species. The aim of this study was to estimate the genetic diversity and population structure of *G. setequedas*, using microsatellite markers and mitochondrial haplotypes, in order to test the hypothesis of low genetic diversity in this restricted population. Muscular tissue samples of 86 specimens were obtained from nine locations in the Lower Iguaçu River basin, between upstream of the Iguaçu Falls and downstream of the Salto Caxias Reservoir. Seven microsatellites loci were examined and a total of 120 different alleles were obtained. The number of alleles per locus (*N*_A_) was 17.429, effective alleles (*N*_E_) 6.644, expected heterozygosity (*H*_E_) 0.675, observed (*H*_O_) heterozygosity 0.592, and inbreeding coefficient (*F*_IS_) 0.128. Twelve haplotypes in the D-Loop region were revealed, with values of *h* (0.7642) and π (0.00729), suggesting a large and stable population with a long evolutionary history. Thus, both molecular markers revealed high levels of genetic diversity and indicated the occurrence of a single *G. setequedas* population distributed along a stretch of approximately 200 km. The pattern of mismatch distribution was multimodal, which is usually ascribed to populations in demographic equilibrium. Nevertheless, the construction of a new hydroelectric power plant, already underway between the Salto Caxias Reservoir and Iguaçu Falls, could fragment this population, causing loss of genetic diversity and population decline, and for this reason it is necessary to maintain the Iguaçu River tributaries and downstream area from the Lower Iguaçu Reservoir free of additional dams, to guarantee the survival of this species.

## Introduction

The largest biodiversity asset in the world is located in Brazil (ICMBio, 2017). However, more than 1,170 species in Brazil have been classified as threatened with extinction. Unfortunately, of these, more than 26% are Actinopterygii fish found in freshwaters (ICMBio, 2017), including *Gymnogeophagus setequedas* Reis et al. (1992), the only one of the 17 species of the genus described so far considered as threatened (Abilhoa and Duboc, 2004; Pavanelli and Reis, 2008). This has led the Brazilian Environmental Ministry to decree the species with the status of Endangered species (EN) (decree #445, International Union for Conservation of Nature [IUCN], 2014).

*Gymnogeophagus setequedas* was described based on specimens collected in tributaries of the Paraná River in Paraguay and Brazil, near the Sete Quedas region, an area currently submerged due to construction of the Itaipu Hydroelectric Power Plant (Reis et al., 1992; Pavanelli and Reis, 2008). However, since its description no specimens have been collected in the original known distribution area (Abilhoa and Duboc, 2004; Agostinho et al., 2004; Pavanelli and Reis, 2008). Nevertheless, 15 specimens of *G. setequedas* were recently collected in the Lower Iguaçu River, both up and downstream from Iguaçu Falls in the Iguaçu National Park (Paiz et al., 2017). According to the authors, finding this species in that region was quite unexpected as the Iguaçu waterfalls have lead to effective geographic isolation of the Ichthyofauna of the Iguaçu River (Zawadzki et al., 1999), providing an accentuated degree of endemicity, estimated between 51 and 71% (Abell et al., 2008). In addition, due to its high ecological importance, the Iguaçu River basin is considered an ecoregion, separated from the rest of the Paraná River Basin (Abell et al., 2008).

The Iguaçu River basin covers an area of approximately 72,000 km^2^, representing part of the landscape of the three Paraná plateaus, subdivided into three regions: Upper Iguaçu (1st plateau, Curitiba region), Middle Iguaçu (2nd plateau, Ponta Grossa region), and Lower Iguaçu (3rd plateau, Guarapuava region) (Maack, 2001). The portion of the 3rd plateau that includes the Lower Iguaçu is characterized by the presence of numerous waterfalls, such as Salto Grande (13 m), Salto Santiago (40 m), Salto Osório (30 m), and Iguaçu Falls (Maack, 1981). The region is very attractive for hydroelectric use due to its high gradient, and, thus, the original rapids and waterfalls, have been transformed into a sequence of reservoirs that flooded approximately 656 km^2^, remarkably altering the landscape (Júlio Júnior et al., 1997).

It was believed that *G. setequedas* preferred lentic environments, as in the other species of the genus (Pavanelli and Reis, 2008). However, it seems that this species behaves differently from its congeners, preferring fast waters. This fact was corroborated by its recent capture in the Lower Iguaçu River, in stretches without containment and with fast waters (Paiz et al., 2017). In addition, this species disappeared after construction of the Itaipu reservoir, being collected only twice, suggesting its dependence on lotic environments (Agostinho et al., 2004). Pavanelli and Reis (2008) consider that this species no longer occurs in the Itaipu reservoir, possibly because it did not succeed in colonizing the environment formed after construction of the reservoir. In Paraguay this species is also considered an EN (Liotta, 2010), for the same reasons as in Brazil.

According to Wu et al. (2015), understanding the diversity and genetic structure of endangered species is fundamental to engage effective environmental conservation and management actions. Genetic diversity is essential if populations are evolving in response to environmental changes. For instance, due to the effects of anthropogenic disturbances, a small and isolated population is more likely to lose genetic diversity, and consequently present population decline, than a huge population with high genetic diversity (Frankham et al., 2010; Allendorf et al., 2012).

The genetic diversity status of species is the starting point for systematic planning of actions that should be taken to ensure the survival of species and reduce their risk of extinction. No works are known which focus on the biological (Pavanelli and Reis, 2008) or genetic diversity of *G. setequedas*. In addition, the diploid number has only recently been presented (Paiz et al., 2017). Thus, the aim of this study was to estimate the genetic diversity and population structure of *G. setequedas* along its recently known area of occurrence, using microsatellite markers and mitochondrial haplotypes (D-loop), thus presenting the first data of a population study of this species threatened with extinction.

## Materials and Methods

### Study Area and Sampling

**FIGURE 1:**
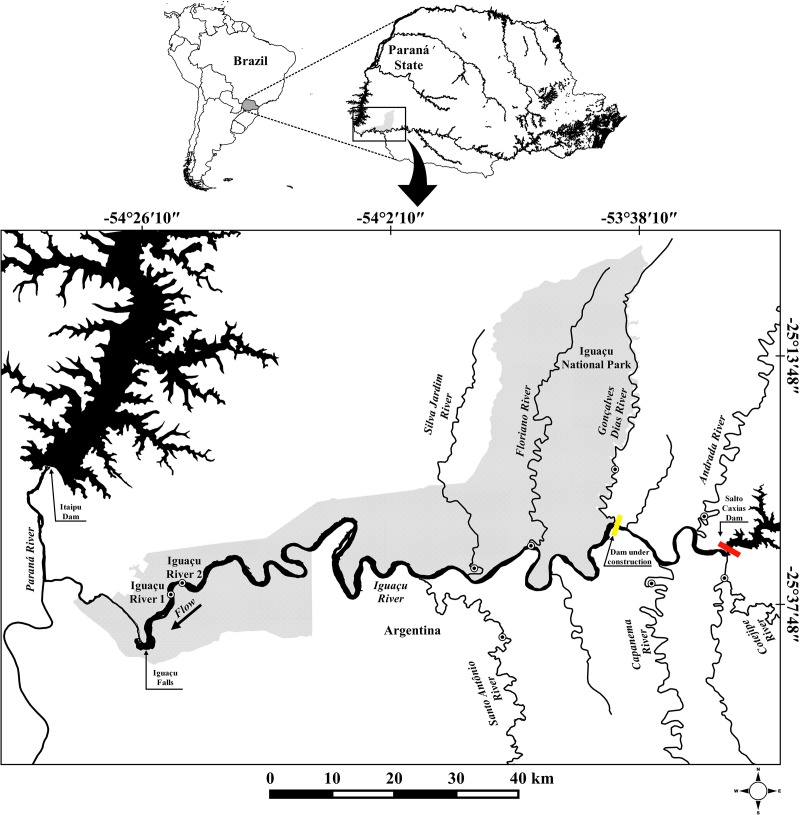
Map of southern South America showing collection points (dotted circle) of *Gymnogeophagus setequedas*. The yellow bar represents the Baixo Iguaçu Hydroelectric Power Plant under construction, and red bar indicates Salto Caxias Hydroelectric Power Plant.

Our study area comprises a stretch of the Lower Iguaçu River basin, between upstream Iguaçu Falls and downstream Salto Caxias Reservoir (**Figure 1**).

Samples of 86 *G. setequedas* were collected at nine different points, some of them located in the Iguaçu National Park (PNI): two points in the main channel of the Iguaçu River (IGU 1 and IGU 2 – PNI), near the Iguaçu falls, and seven tributaries of the Iguaçu River (STO-Santo Antônio, SIL-Silva – PNI, Jardim, FLO-Floriano – PNI, GON-Gonçalves Dias – PNI, CAP-Capanema, AND-Andrada, and COT-Cotegipe) (**Figure 1** and **Table 1**). The samples were collected in 2012 (November), 2013 (November and December), and 2014 (January, February, March, April, July, August, September, November, and December). The specimens were captured using nets of different mesh sizes and electric fishing. Samples of muscle and rayed fins were taken from the fish, stored in microtubes containing 100% ethanol and kept at -20°C. Specimens were fixed in 10% formalin and preserved in 70% ethanol and deposited in the fish collection of the Zoology Museum at the Universidade Estadual de Londrina under catalog number: MZUEL 16332, 16353, 16354, 17094–17096.

**Table 1:** Sampling sites for *Gymnogeophagus setequedas*, including sample sizes per site (N) and location.

Collection point	*N*	Specific collecting location	Geographic coordinates
(1) – (IGU 1)	02	Iguaçu River 1	25°35′43.79^′′^S 54°22′17.51^′′^W
(2) – (IGU 2)	50	Iguaçu River 2	25°36′50.77^′′^S 54°23′23.42^′′^W
(3) – (STO)	25	Santo Antônio River	25°40′57.60^′′^S 53°51′22.91^′′^W
(4) – (SIL)	01	Silva Jardim River	25°34′18.64^′′^S 53°54′06.13^′′^W
(5) – (FLO)	01	Floriano River	25°32′08.12^′′^S 53°48′37.46^′′^W
(6) – (GON)	02	Gonçalves Dias River	25°24′45.53^′′^S 53°40′30.30^′′^W
(7) – (CAP)	01	Capanema River	25°35′46.76^′′^S 53°36′58.98^′′^W
(8) – (AND)	03	Andrada River	25°29′18.17^′′^S 53°31′54.16^′′^W
(9) – (COT)	01	Cotegipe River	25°35′15.39^′′^S 53°29′57.11^′′^W

### DNA Extraction and Quantification

Total DNA was extracted from muscle or rayed fins preserved in 95% EtOH following the phenol/chloroform protocol of Almeida et al. (2001). NanoDrop^TM^ 1000 was used for determination of DNA concentrations and samples were diluted in ultrapure water, 10 ng/μL for microsatellite markers and 5 ng/μL for mtDNA D-loop markers.

### Microsatellite Amplification and Genotyping

Cross-amplification tests were conducted from seven loci described for *Geophagus brasiliensis* (Gbra6, Gbra16, Gbra17, Gbra62, Gbra63, Gbra80, and Gbra96) (Ferreira et al., 2013). Reagent concentrations and PCR conditions were performed according to Ferreira et al. (2015), using the modifications proposed by Schuelke (2000). PCR thermal conditions were conducted as follows: initial denaturation step at 94°C for 4 min, followed by 35 cycles of denaturation at 94°C for 40 s. The annealing temperatures of successful cros-amplifications loci were 48°C (Gbra16, Gbra62, Gbra63, Gbra80), 54°C (Gbra06, Gbra17, Gbra70), or 60°C (Gbra96) for 1 min, extension at 72°C for 1 min, followed by a final extension at 72°C for 30 min. PCR products were analyzed on an ABI PRISM 3500-XL automated sequencer (Applied Biosystems) using GeneScan 600 Liz (Applied Biosystems) as a molecular weight marker.

### mtDNA (D-Loop) Marker

Part of the control region (D-loop) of the *G. setequedas* mitochondrial DNA was amplified using PCR. The primers used were L 5′-AGAGCGTCGGTCTTGTAAACC-3′ (Cronin et al., 1993) and H 5′-CTGAAGTAGGAACCAGATG-3′ (Meyer et al., 1990). PCR reactions were performed in a 25 μL final volume containing 1X GoTaq Master Mix (Promega), 1 μM of each primer, 15 ng DNA, and ultrapure water to volume. The thermal profile included an initial denaturation at 94°C for 4 min, followed by 41 cycles at 94°C for 15 s, annealing at 56°C for 30 s, and extension at 72°C for 2 min, with a final extension at 72°C for 10 min. The PCR products were purified using ExoSAP IT (Prodimol Biotecnologia S.A, Belo Horizonte, Minas Gerais, Brazil). The Big Dye Terminator v 3.1 kits (Applied Biosystems) and ABI-PRISM 3500 XL automated sequencer (Applied Biosystems) were used for sequence analysis. Multiple alignment analysis was carried out using the ClustalW application (Thompson et al., 1994) in BioEdit 7.1.3.0 (Hall, 1999). NCBI’s BLAST search (Basic Local Alignment Search Tool, Altschul et al., 1990) was used to confirm the origin of the fragment. To search for possible tRNA, was used an online version of the tRNAscan-SE (Lowe and Eddy, 1997), available at http://lowelab.ucsc.edu/tRNAscan-SE. Sequences of the 12 different haplotypes were deposited in GenBank (MG581478 to MG581489).

### Genetic Analyses (Microsatellites)

#### Population Structure

The first step for genetic analyses was to define the number of existing populations. For this we used population analyzes based on Bayesian approaches that mainly include “attribution methods.” These methods calculate the probability of the different genotypes being observed in each population and assign the individuals to the populations according to the possibilities of the genotypes belonging to them, without any a priori inference. Thus, such analyzes allow to infer which population an individual belongs to, regardless of their collection site (Beaumont and Rannala, 2004).

In order to evaluate the relationship between samples, we conducted a Bayesian cluster analysis of the population by using STRUCTURE v.2.3.3 (Pritchard et al., 2000) program. The number of populations (*K*) was estimated by using the admixture model and correlated allele frequencies among populations, with *K* ranging from 1 to 10 (*K* = 1–10) (Evanno et al., 2005). A total of 20 independent runs of 100,000 Markov Chain Monte Carlo (MCMC) iterations discarded as burn-in, followed by 1,000,000 MCMC iterations were used for each value of *K*. The best-fit number of groupings was evaluated using *K*, ln Pr (X/*K*) (Pritchard et al., 2000) and Δ*K ad hoc* statistics (Evanno et al., 2005) by Structure Harvester v.0.6.7 (Earl and VonHoldt, 2012). Graphs representing the membership coefficient of each sampled individual were plotted using Distruct 1.1 (Rosenberg, 2004). Genetic differentiation estimates were assessed from pairwise Φ_ST_ values obtained in ARLEQUIN v.3.5.1.3 (Excoffier and Lischer, 2010). Significant estimates were based on 10,000 permutations. Subsequently, *P*-values corresponding to alpha = 0.05 were adjusted after Holm-Bonferroni correction for multiple tests (Holm, 1979).

### Genetic Diversity

Number of alleles per locus (*N*_A_), effective number of alleles (*N*_E_), expected and observed heterozygosity (*H*_O_, *H*_E_) were obtained with POPGEN v. 1.31 (Yeh et al., 2000) software. Inbreeding coefficient (*F*_IS_) was obtained with Fstat v2.9.3 program (Goudet, 2001). Deviation from Hardy-Weinberg equilibrium (HWE) and the linkage disequilibrium between pairs of loci with significance (*P*-value), later adjusted by the Bonferroni sequential correction (Rice, 1989) were tested with the GENEPOP v.1.2 (Raymond and Rousset, 1995). MICRO-CHECKER 2.2.1 (Van Oosterhout et al., 2004) software was used to test for the possible presence of null alleles or other genotyping errors such as allelic dropout and reading errors due to stutter peaks.

### Gene Flow

The contemporary migration rates over a few previous generations and the direction of migration among the samples studied, was estimated by using the BayesAss v 3.0.3 program (Wilson and Rannala, 2003), at 95% confidence intervals. Ten runs were analyzed using different random starting seed numbers, with 3,000,000 MCMC iterations, including 999,999 discarded burn-in iterations. After the burn-in, every 2000th iteration was sampled. The delta values (maximum amount by which parameter values are allowed to change between iterations) were 0.15 for allele frequencies, 0.025 and 0.05 for migration rate, and 0.15 for inbreeding value.

### Demographic Analyses

Recent population bottleneck signs were evaluated on microsatellite data using Bottleneck v.1.2.02 program (Piry et al., 1999), considering deviations from the mutation-drift equilibrium. Three tests were used, including two tests to indicate bottlenecks in the presence of significant excess heterozygosity: “Sign test” (Cornuet and Luikart, 1996) and the “Wilcoxon sign-rank test” (Luikart and Cornuet, 1998), both based on the Infinite Alleles Model (IAM), Stepwise Mutation Model (SMM), and Two-Phase Model (TPM – with 90% SMM and 10% IAM), with a *P*-value < 0.05. The third test was the “Mode shift test” that indicates bottlenecks resulting from alterations in allele frequency distributions (Luikart et al., 1998).

### Genetic Analyses (mtDNA)

#### Genetic Diversity

Haplotype number, haplotype diversity (*h*) and nucleotide diversity (π) were obtained from DnaSP v.5 program (Librado and Rozas, 2009). Arlequin v.3.5.1.3 program (Excoffier and Lischer, 2010) was used to conduct the selective neutrality tests based on the infinite sites model of Tajima (D) (Tajima, 1989) and Fu (Fs) (Fu, 1997). The Network v.4.6.1.1 program (Fluxus Technology Ltd.^1^) was used to construct haplotype networks from mtDNA data based on the median-joining algorithm (Bandelt et al., 1999).

## Results

### Population Structure

**FIGURE 2:**
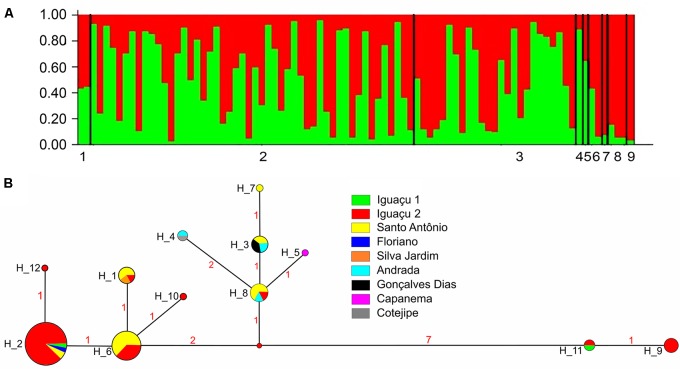
**(A)** Graphical representation of Bayesian cluster analysis from *K* = 1. Each column represents a different individual of *G. setequedas*, and the colors represent the probability of the ancestral coefficient of individual in each genetic cluster. Numbers represent the collection points: (1) Iguaçu 1; (2) Iguaçu 2; (3) Santo Antônio; (4) Silva Jardim; (5) Floriano; (6) Gonçalves Dias; (7) Capanema; (8) Andrada; (9) Cotegipe. **(B)** Haplotype network based on partial sequencing of the D-loop region (mtDNA) of 82 individuals of *G. setequedas* from the Lower Iguaçu River. Circle sizes are proportional to haplotype frequency. Numbers between haplotypes denote mutational steps between sequences.

Bayesian clustering analysis (Structure) applied to microsatellite data indicated that the most probable *K* (*K*+ cluster number) was *K* = 1, from ln Pr(*X*/*K*). The graphic representation of Δ*K* showed that there were no well-defined groups, the ancestral values were distributed homogeneously among individuals and samples, indicating the occurrence of a single *G. setequedas* population (**Figure 2A**). Molecular Variance Analysis (AMOVA), conducted for all samples showed very little variation among them (Φ_ST_ = 0.025, *P* > 0.05). Following this result, all other analyzes were based on a single population.

### Genetic Diversity

In the entire sample, a total of 120 different alleles were obtained from seven microsatellite loci. The number of alleles per locus (*N*_A_) was 17.429, effective alleles (*N*_E_) 6.644, expected heterozygosity (*H*_E_) 0.675, observed (*H*_O_) heterozygosity 0.592, and inbreeding coefficient (*F*_IS_) 0.128.

After applying the Bonferroni sequential correction, there were no significant deviations (*P* < 0.05) in the Hardy–Weinberg equilibrium (HWE) at the majority of microsatellite loci, only the locus *Gbra*96 showed significant deviation. This correction was also applied to the linkage disequilibrium (LD) tests, and a significant value was found only in locus *Gbra*96. The Micro-Checker program found no null alleles among the samples.

From the amplification and sequencing of mtDNA of 82 *G. setequedas* individuals, a 449 bp fragment from the D-loop region was obtained. Twenty polymorphic sites (17 transition and three transversion mutations) and four indels sites were found. Twelve different haplotypes were revealed, of which four haplotypes (H5, H7, H10, and H12) were singletons (**Figure 2B**). The H2 haplotype was the most frequent, observed in 37 samples from four different locations (IGU1, IGU2, STO, and FLO). Although SIL, FLO, GON, COT, and CAP present only one haplotype each, these haplotypes are shared with other locations, except for H5 found only in CAP. The IGU2 has the highest number of haplotypes (*N* = 8), followed by STO (*N* = 6), AND (*N* = 3), and IGU1 (*N* = 2). Haplotype (*h*) and nucleotide (π) diversity values were 0.7642 and 0.00729, respectively (**Table 2**).

**Table 2:** Genetic diversity of *G. setequedas* in the Lower Iguaçu River basin, based on microsatellite markers and mitochondrial haplotypes (D-Loop).

Microsatellites							mtDNA					
*N*	*A*	*N*_A_	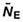	*H*_O_	*H*_E_	*F*_IS_	*N*	Nh	*h*	π	*D*	*F*_S_
86	120	17.143	6.574	0.593	0.673	0.126	82	12	0.7642	0.00729	-0.570	-0.285

*N, number of individuals examined; A, total number of alleles; N_A_, mean number of alleles; N_E_, mean number of effective alleles; H_O_, observed heterozygosity; H_E_, expected heterozygosity; F_IS_, inbreeding coefficient; Nh, number of haplotypes; h, haplotype diversity; π, nucleotide diversity; D, Tajima’s neutrality test; Fs, Fu’s neutrality test.*

### Demographic Analyses

**FIGURE 3:**
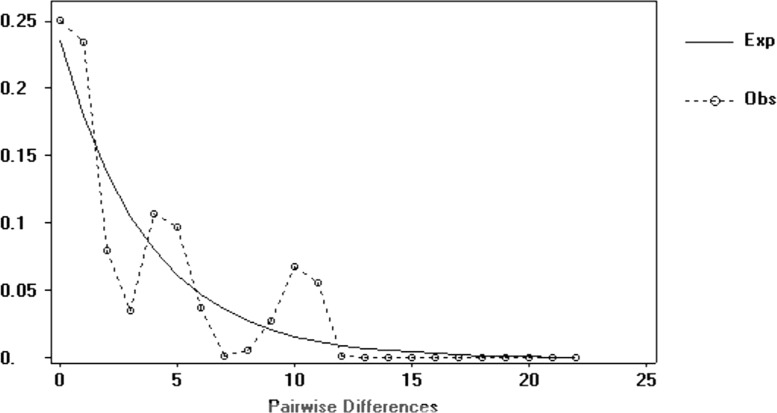
Mismatch distribution of mitochondrial haplotypes obtained from *G. setequedas* samples studied in the Lower Iguaçu River basin.

The signed-rank test did not produce significant values in any of the mutational models (IAM, SSM, or TPM). In the mode-shift test, the samples showed typical L-shaped distribution (non-bottleneck) in the frequency of the alleles in the mode-shift test (**Table 3**). The mismatch distribution graphic demonstrated a multimodal distribution for haplotypes (**Figure 3**), which is usually ascribed to populations in demographic equilibrium. In the neutrality tests, Tajima test values (*D*) and Fu test values (*F*_s_) were negative and not significant (**Table 2**).

**Table 3:** Bottleneck tests in 86 samples of *G. setequedas* from the Lower Iguaçu River Basin.

		Sign test/Wilcoxon sign-rank test						Allele frequency distribution
		IAM^a^		TPM^b^		SMM^c^		
	*N*	*H*_d_/*H*_e_	*P*	*H*_d_/*H*_e_	*P*	*H*_d_/*H*_e_	*P*	
Total	86	3/4	0.710	6/1	0.996	7/0	1.000	*L-shaped*

*N, number of individuals; H_e_, number of loci exhibiting excess heterozygosity; H_d_, number of loci exhibiting deficient heterozygosity. Normal L-shaped distribution = non-bottlenecked population. ^a^Infinite allele model, ^b^two phase model, ^c^stepwise mutation model.*

### Gene Flow

Bayesian gene flow analysis of the microsatellite data revealed contemporary migration values among the samples within the confidence interval (95%). Values for non-immigrants in each sample, ranging from 79 to 82.6%. However, migration estimates were also obtained and showed similar values among the majority of samples. The lowest migration estimates were from IGU 1 to COT and IGU 2 to AND, both with 1.9%. On the other hand, the highest migration values were from FLO to IGU2, and to GON, and COT to CAP, with 2.7%. Among all the samples, FLO was the one that obtained the highest percentage of migrants, for the largest number of sites (**Table 4**).

**Table 4:** Gene flow estimates based on Bayesian inferences of migration rates using BayesAss.

Migration	IGU 1	IGU 2	STO	SIL	FLO	GON	CAP	AND	COT
IGU 1	**82.6%**	2.2%	2%	2.1%	2.6%	2.4%	2.4%	2.4%	2.4%
IGU 2	2.1%	**82.2%**	2.2%	2.1%	2.7%	2.2%	2.5%	2.5%	2.5%
STO	2%	2.3%	**81.5%**	2.3%	2.3%	2.4%	2.4%	2.4%	2.4%
SIL	2.2%	2.4%	2.5%	**81.7%**	2.6%	2.4%	2.4%	2.4%	2.4%
FLO	2%	2.2%	2.2%	2.3%	**79%**	2.4%	2.6%	2.4%	2.5%
GON	2.2%	2.2%	2.4%	2.3%	2.7%	**80.7%**	2.6%	2.5%	2.6%
CAP	2.2%	2.2%	2.4%	2.2%	2.5%	2.4%	**79.7%**	2.6%	2.7%
AND	2.4%	1.9%	2.1%	2.4%	2.5%	2.4%	2.3%	**80%**	2.2%
COT	1.9%	2%	2.2%	2.1%	2.6%	2.4%	2.5%	2.4%	**79.8%**

*The bold values along the diagonal represent non-migrants within a putative source subpopulation.*

## Discussion

### Genetic Diversity and Population Structure

The study of molecular markers, such as Microsatellite and mtDNA, generates important information on the genetic variation and structure of fish species and is a significant step toward realizing the goal of conservation of species in their natural populations (Carvalho-Costa et al., 2008; Piorski et al., 2008; Garcez et al., 2011; Abdul-Muneer, 2014). According to Wu et al. (2015), understanding the diversity and genetic structure of endangered species are essential to engage effective environmental conservation and management action. The long-term persistence of species depends on sufficient genetic diversity to adapt and survive in variable or changing environments (Hughes et al., 2008).

According to DeWoody and Avise (2000), based on a meta-analysis of microsatellite polymorphisms, freshwater fish, on average, have 9.1 ± 6.1 alleles and expected heterozygosity of 0.54 ± 0.25 per population. Therefore, based on microsatellites, the genetic diversity of the *G. setequedas* population in terms of allele numbers (17.14) and the expected heterozygosity (0.67) are as expected for freshwater fish. In addition to the high diversity in the nuclear markers, the *G. setequedas* analyzed showed significant variations in mitochondrial DNA, exhibiting high levels of genetic diversity. According to Freeland (2005), *h* is considered the haploid equivalent of HE in data on diploids. The similarity among these estimates suggests that the current variations in nuclear and mitochondrial DNA are evenly distributed throughout *G. setequedas* population.

A high level of genetic diversity is an important attribute for species and may confer the basis for adaptation to environmental change (Piorski et al., 2008), especially when it comes to endangered species such *as G. setequedas* (Abilhoa and Duboc, 2004; Pavanelli and Reis, 2008; International Union for Conservation of Nature [IUCN], 2014).

### Demographic History

A 449 bp test in the D-Loop region, one of the most variable regions of mtDNA (Frankham et al., 2010), revealed 12 haplotypes and high values of π (0.00729) and *h* (0.750). In addition, analysis of the microsatellite data using Bottleneck program showed no significant recent bottlenecks. The absence of recent bottlenecks is corroborated by the high haplotype (*h* > 0.5) and nucleotide diversity (π > 0.5%) values in the mtDNA. According to Grant and Bowen (1998), high haplotypic diversity combined with high nucleotide diversity represents a large and stable population with a long evolutionary history, or secondary contact between different lineages. Stable population with a long evolutionary history seems to be a very plausible possibility for *G. setequedas*, as the Iguaçu waterfalls have exerted effective geographic isolation on ictiofauna of the Iguaçu river (Zawadzki et al., 1999), providing an accentuated degree of endemicity, of more than 70% (Abell et al., 2008). In addition, analysis of the distribution of substitution differences between pairs of haplotypes (mismatch distribution) (Cunha and Solé-Cava, 2012) shows multimodal distributions, which is generally attributed to populations in demographic equilibrium (Rogers and Harpending, 1992).

However, negative values in the Tajima’s *D* test and Fu’s *F*_s_ test, even if not significant, could suggest population expansion after an ancient bottleneck (Slatkin and Hudson, 1991; Grant and Bowen, 1998), indicating that all the current haplotypes are closely related and derived from a single main haplotype (H2). Signs of old bottlenecks may be less evident at microsatellite loci, since they tend to recover from the variation more rapidly than mitochondrial sequences. At the same time, π recovery after a genetic bottleneck is slower than *h* at mtDNA (McCusker and Bentzen, 2010).

### Gene Flow

The individuals of the Floriano River (FLO) presented the highest rates of migration, and the highest levels of admixture in samples were found in the Iguaçu 2 and Gonçalves Dias rivers. The specimens from rivers further upstream in the drainage (FLO, GON, CAP, and COT) appear to have more levels of admixture between them. This factor might suggest that entry into the upper tributaries is more likely than the lower. However, the highest rates of migrants to Iguaçu 1 are from most upstream tributaries, suggesting that populations of *G. setequedas* maintain satisfactory gene flow in all stretches of the river studied. According to Palstra and Ruzzante (2008), if local populations are small, as is the case in the present study, gene flow is the key factor to prevent the stochastic loss of genetic diversity, besides providing the required alleles to subpopulations under selection that lack favorable genotypes (Kinnison and Hairston, 2007). Although these results allow inferring gene flow between localities, according to Wilson and Rannala (2003) a strong estimate can be reach with a higher sample size per locality. It can be the next goal for further studies, but it is a difficult task to solve immediately because the species is not abundant and is mainly distributed in a preservation area.

According to Fagan et al. (2002, 2005), riverine populations are forecasted to be particularly vulnerable to fragmentation due to their dendritic structure, which may be exacerbated by unidirectional migration. Natural barriers (rapids and waterfalls) and man-made structures, such as dams, also fragment riverine populations, influencing in the dispersal rate and migration pattern (Wofford et al., 2005), even of a rheophilic species of fishes with strong swimming abilities such as *G. setequedas* (Paiz et al., 2017). However, the construction of a new hydroelectric power plant (Baixo Iguaçu HPP), already underway between the Salto Caxias Reservoir and Iguaçu Falls, could fragment this population preventing the gene flow. As a consequence, there may be loss of genetic diversity and population decline, especially in the area of future reservoir. Moreover, this separate population can be extinguished, as has already happened with another population of *G. setequedas* after the construction of the Itaipu Hydroelectric Power Plant. The disappearance was attributed to the lentic waters of the Itaipu Reservoir, which isolated populations of this rheophilic species, which previously occurred in tributaries of both river banks, in Paraguay and Brazil, and probably in the Paraná River (Paiz et al., 2017).

### Conservation Implications

The abundance, dispersal, and population size are reduced in populations structured by habitat fragmentation due to barriers such as dams, thereby increasing the risk of extinction (Gross et al., 2004; Letcher et al., 2007). This fragmentation can lead to the total or partial isolation of a population, conditioning the response of the individuals. Thus, in the recently found population of *G. setequedas* in the Iguaçu River, a drastic reduction and loss of genetic diversity, due to inbreeding, must be avoided preserving the lotic characteristics of the environment.

For instance, due to the effects of anthropogenic disturbance, small and isolated populations are more likely to suffer loss of genetic diversity and population decline, than a huge population with high genetic diversity (Frankham et al., 2010; Allendorf et al., 2012). According to Frankham (2003), inbreeding reduces reproduction and survival rates, and loss of genetic diversity reduces the ability of populations to evolve to cope with environmental changes, leading to extinction risk.

The type locality and most of the records of *G. setequedas* are in Paraguay, in tributaries of the right bank of the Paraná river, in the region of influence of the Itaipu reservoir and downstream (Reis et al., 1992). Since the species description, despite several attempts, it was not possible to collect new specimens from the known geographic range of occurrence (Agostinho et al., 2004; Pavanelli and Reis, 2008). According to Pavanelli and Reis (2008), this species no longer occurs in the Itaipu reservoir, as well as in the floodplain upstream of the reservoir. Despite several collection efforts on the Iguaçu River (Pavanelli and Reis, 2008), mainly in the Lower Iguaçu upstream the National Park (Baumgartner et al., 2012), this species was not collected. For this reason, the conservation status of *G. setequedas* was invariably attributed to a threatened category (Abilhoa and Duboc, 2004; Pavanelli and Reis, 2008; International Union for Conservation of Nature [IUCN], 2014; Paiz et al., 2017). However, recently Paiz et al. (2017) and the present study report the presence of *G. setequedas* in the Lower Iguaçu in the National Park region. In this way, the population of *G. setequedas* of the Lower Iguaçu River may be one of the last remnants of this species and, according to Pavanelli and Reis (2008), as *G. setequedas* is a naturally rare species, it is advisable that any anthropogenic changes in its original ecosystem be discouraged.

The results presented here demonstrate that the population of *G. setequedas* of the Iguaçu River still maintains satisfactory levels of genetic diversity. However, in terms of conservation management plans, to guarantee the survival of this species, it is necessary to maintain the tributaries of the Iguaçu River and the downstream area from the future reservoir (Baixo Iguaçu Reservoir) without additional dams. Long-term monitoring of genetic diversity and inbreeding could also help conserve this population and provide a basis for future decisions.

## Ethics Statement

This study was carried out in strict accordance with the recommendations provided in the Guide for the Care and Use of Laboratory Animals. Collection was authorized by the System of Authorization and Information on Biodiversity – SISBIO (SISBIO n°. 25648-3 and 25648-4), by the Chico Mendes Institute for Biodiversity Conservation ICMBio 003/2014 and Official SEI n°. 63/2016-DIBIO/ICMBio), and by the Environmental Institute of Paraná – IAP (n°. 37788 and 43394). The sampling protocol was approved by the Ethics Committee on the Use of Animals – CEUA of the Universidade Estadual do Oeste do Paraná (n°. 62/09).

## Author Contributions

LS-S, DF, MM, and OS designed the research. LA, SP, SM, and MM collected data. LS-S, TK-D, and DF performed the molecular genetic studies. All authors contributed to the writing of the manuscript.

## Conflict of Interest Statement

The authors declare that the research was conducted in the absence of any commercial or financial relationships that could be construed as a potential conflict of interest.

## Footnotes

1 http://www.fluxus-engineering.com
